# To travel is to live: embracing the emerging field of travel psychiatry

**DOI:** 10.1192/bjb.2020.32

**Published:** 2021-06

**Authors:** Gerard Flaherty, Shang Yuin Chai, Brian Hallahan

**Affiliations:** 1 School of Medicine, National University of Ireland Galway, Ireland; 2 School of Medicine, International Medical University, Kuala Lumpur, Malaysia; 3 Galway Roscommon Mental Health Services, University Hospital Galway, Ireland

**Keywords:** Travel, risk assessment, psychosis, suicide, stigma and discrimination

## Abstract

For a person with mental illness, travelling abroad can be challenging but it can be easier when the traveller and healthcare practitioner have a clear understanding of the likely impact of travel on the illness and of the illness on the travel experience. Travel may also precipitate first presentations of mental illness or unmask previously undiagnosed mental disorders. We propose that mental health problems should receive greater recognition in travel medicine and that psychiatrists should collaborate more closely with travel medicine clinicians to ensure that their patients benefit from the opportunities afforded by international travel.

‘To move, to breathe, to fly, to float, to gain all while you give, to roam the roads of lands remote, to travel is to live.’

Hans Christian Anderson, *The Fairy Tale of My Life: An Autobiography* (1847)

The psychological benefits of international travel, especially for tourism purposes, have been largely ignored until relatively recently in the travel medicine literature, whose focus to date has been on the mitigation of travel health risks through vaccination and pre-travel physical health counselling. The unique challenges faced by travellers with diagnosed mental illness are rarely addressed in the pre-travel medical consultation. Furthermore, travel may precipitate first clinical episodes of mental illness in an unfamiliar environment. Travel medicine physicians without basic knowledge of psychiatry are often poorly prepared to anticipate the likelihood of decompensation of mental illness in this cohort of travellers. We propose travel psychiatry as an emerging area of both research and clinical interest. This article draws on the sparse available literature and over 40 years of combined clinical experience in psychiatry (B.H.) and travel medicine (G.F.) to describe the effects of international travel, both positive and negative, on people with mental illness, with the aim of advocating for an integrated approach to supporting individuals with mental illness when travelling abroad.

## Mental health challenges of international travel

Travel, even with a recreational motivation, is inherently stressful and the perceived stressful impact is likely a product of the travel itinerary, destination, activities and individual traveller susceptibility. Felkai & Kurimay reported that 11.3% of travellers experience some symptoms of mental illness during travel, with 0.3% of travellers experiencing an acute psychotic episode.^1^

Indeed, incidence data reveal that acute psychotic episodes account for one-fifth of travel-related mental illnesses. Travellers who are deemed medically fit to travel and who have no history of mental disorder may develop acute *de novo* psychosis during their trip. The cumulative effects of travel-induced stress, culture shock, inappropriate alcohol intake and/or recreational substance use, circadian rhythm disruption, underlying brain pathology and physical illness have been implicated as contributing factors in the development of first-time psychosis during travel.^1–3^ Another subgroup of travel-related psychosis occurs during travel to destinations with high religious, cultural and aesthetic value, so-called high-valence venues.^3^ However, many of these episodes occur against a background of a pre-existing psychotic illness. Airault & Valk describe a number of specific destination syndromes, including Jerusalem syndrome, Stendhal syndrome and Paris syndrome.^3^ In addition, travel to high-altitude destinations is increasingly regarded as an additional risk factor for the development of acute psychotic episodes and an increased risk of both suicidal ideation and completed suicide.^4^

Misuse of alcohol and/or psychoactive substances among international travellers may be associated with an exacerbation of existing mental illness and could precipitate an acute psychotic episode.^1,5^ Physicians should consequently be mindful about their patients travelling abroad for the purpose of seeking therapeutic or recreational drugs. Drug tourism, whereby tourists cross international borders for the purpose of obtaining or using psychoactive substances, is associated with significant adverse health effects, including drug-induced psychosis, unintentional physical injury, risky sexual behaviour and criminal acts.^5^ In addition to drugs of misuse, medications used in travel medicine (such as mefloquine for malaria chemoprophylaxis) can potentially trigger acute psychotic episodes in people with a previous or undiagnosed mental illness.^1,5^

Anxiety symptoms are common in individuals engaging in travel. The sense of uncontrollability and inherent unpredictability presented by travel may cause travellers to develop anxiety symptoms or worsen an existing anxiety disorder. Culture shock is a travel phenomenon that exposes travellers to the risk of anxiety symptoms, in addition to confusion, a sense of isolation, rejection and deprivation due to cultural differences experienced while abroad. Patients may feel overwhelmed when immersed in an unfamiliar destination and struggle to make the necessary adaptations to acclimatise to local customs and cultural norms. Moreover, travelling abroad inevitably leads to a lack of daily structural routine, separation from social supports in some cases and a lack of support, which can precipitate poor adherence to prescribed psychotropic medications. Furthermore, there is evidence that jet lag can exert a debilitating effect on travellers with mental illness, with sleep loss being demonstrated to increase the risk of episodes of (hypo)mania in individuals diagnosed with bipolar disorder.^2^ This may be significant in international travel if the patient encounters circadian rhythm disruption and other stressors that result in sleep disturbance.

Where an individual develops an acute episode of mental illness, receiving appropriate intervention in a timely fashion can be challenging for a number of reasons, including problems communicating health difficulties to clinicians, cultural differences, poorly established diagnoses and lack of clarity in relation to the psychiatric history of the traveller. Furthermore, difficulties may arise if there is delay in the diagnosis of physical illness, such as hypoglycaemia, epileptic aura or hypothyroidism, which may initially present with alterations in mental health. Repatriation to the home country where patients can receive specialised treatment from their multidisciplinary mental health team in a familiar environment may be the most appropriate intervention.^1^ However, repatriation for psychiatric reasons can be a particularly complex undertaking requiring, in many cases, medical escort by a suitably trained mental health professional(s) and stabilisation of the person's mental health before costly aeromedical evacuation that may not be covered by travel health insurance.^6^

## Is travel advisable for people with mental illness?

Although it is reasonable to postulate that leisure activities abroad may be beneficial for people with mental illness, research is lacking in this area. One qualitative study from The Netherlands explored how engaging in travel contributes positively to the mental rehabilitation of psychiatric patients.^7^ Material was collected from participant observation involving eleven travellers with severe and enduring chronic mental illness who were accompanied by four psychiatric nurses during two trips. Many positive experiences were reported, including maintenance of social contact; the opportunity to develop foreign language skills; enrichment of life resulting from enjoyment and cherishable memories; positive influence on self-esteem; and a departure from the monotonies of daily routine life.^7^ The research also provided a learning environment outside of psychiatric institutions for the nurses involved. Some travellers required the nurses’ support in managing psychotropic medications and some activities of daily living. We have recently reported similar positive findings from semi-structured interviews conducted with a series of patients with chronic psychotic illnesses who engaged in travel independently, on their own or with friends.^8^ The putative mental health benefits of ‘prescribed travel’ should be balanced against the risks in less-supported, lone travellers of developing suicidal ideation or experiencing exacerbation of mental illness. This may potentially arise from unexpected travel-related stressors, including, for example, confrontations at airport security stations.

## Travel mental health recommendations

Travel psychiatry has a promising future as an integrated subspecialty of both psychiatry and travel medicine. Novel research will help to establish a stronger evidence base for clinical recommendations aimed at promoting mental health during travel.^9^ We recommend that clinicians be aware of the psychological stress and exacerbating factors that patients encounter during foreign travel and encourage patients to attend a pre-travel medical consultation in order to agree on preventive strategies (Box 1).

Box 1 Preventive strategies in travel psychiatry: mental health recommendations for patients and clinicians

Pre-travel

- Attend a pre-travel medical consultation, where stressful events that might be encountered during travel can be considered
- Identify significant risk factors (e.g. psychiatric history, purpose of travel, destination of travel, prescribed medications)
- Obtain comprehensive travel health insurance
- Carry a brief letter from healthcare provider about psychiatric history
- Research the destination and health facilities available locally
- Schedule a pre-travel psychiatric assessment
- Reconsider daily dosages of existing psychotropic medications

During travel

- Educate traveller about jet lag effects
- Ideally, travel with family or friends
- Prescribe a psychotropic drug with anxiolytic properties during the flight, e.g. a low-dose antipsychotic or hypnotic, but avoid benzodiazepines
- Remain in contact with medical practitioner through email or web call
- Encourage treatment adherence with support from travelling companion
- Have a regular, well-planned schedule
- Avoid ‘triggers’ of mental illness
- Avoid psychoactive substance use and caution with use of alcohol
- Seek medical help if a deterioration in mental health occurs

Post-travel

- Follow up with healthcare professional
- Post-travel psychiatric assessment

Suicide tourism, be it planned or unplanned suicide, is one of the leading causes of mortality among international travellers.^10^ Research, including psychological autopsies, should focus on investigating risk factors for suicidal ideation during international travel in an effort to increase the detection of such motivations in intending travellers, who may not consult a travel clinic or psychiatrist in advance of travel.

Post-travel psychiatric assessment may be indicated for certain vulnerable traveller groups, such as volunteers and humanitarian aid workers exposed to hostile and psychologically challenging environments, to counter the effects of post-traumatic stress disorder.^11^ Consideration should also be given to the possibility of reverse culture shock, where travellers experience depressive symptoms and disorientation on returning to their home country.

Routine coverage of psychiatric disorders in travel insurance policies should be ensured in order to provide financial security to people with mental illness travelling abroad. Unfortunately, travellers with pre-existing mental illness often find themselves excluded from such policies and this greatly complicates efforts at their repatriation.

## Conclusions

The lack of research and clinical attention given to travel-related mental health benefits and risks merits the development of an integrated subspecialty of travel psychiatry. This would advance our understanding of the interaction of travel and mental illness in a variety of traveller groups and travel settings. Travel psychiatry should involve close cooperation between mental health professionals and travel medicine practitioners, with ample scope for collaborative patient-centred research. Travel medicine practitioners should receive enhanced training on the psychiatric aspects of travel: travel-related psychological stressors, the impact of travel on pre-existing disorders and the potential for first-time episodes of mental illness during travel, as well as the importance of timely consultation with a travel psychiatrist.

## About the authors

**Gerard Flaherty** is a professor at the School of Medicine, National University of Ireland Galway, Ireland and an adjunct professor at the School of Medicine, International Medical University, Kuala Lumpur, Malaysia. **Shang Yuin Chai** is a final year medical student at the School of Medicine, National University of Ireland Galway, Ireland. **Brian Hallahan** is a senior lecturer at the School of Medicine, National University of Ireland Galway and a consultant psychiatrist at the Galway Roscommon Mental Health Services, University Hospital Galway, Ireland.
